# Extractive-liquid sampling electron ionization-mass spectrometry (E-LEI-MS): a new powerful combination for direct analysis

**DOI:** 10.1038/s41598-023-33647-5

**Published:** 2023-04-20

**Authors:** Adriana Arigò, Giorgio Famiglini, Nicole Marittimo, Marco Agostini, Caterina Renzoni, Pierangela Palma, Achille Cappiello

**Affiliations:** 1 LC-MS Lab, Department of Pure and Applied Sciences, University of Urbino Carlo Bo, Piazza Rinascimento 6, 61029 Urbino, Italy; 2 Laboratorio di Tossicologia, A.S.T. AV1, Via Lombroso 15, 61122 Pesaro, Italy; 3 Department of Chemistry, Vancouver Island University, B-360-R306, 900 Fifth St., Nanaimo, BC Canada

**Keywords:** Analytical chemistry, Environmental chemistry, Green chemistry

## Abstract

One of modern analytical chemistry main challenges is providing as fast as possible results in different application fields. In this view, real-time analysis techniques are experiencing ever-increasing success as they can provide data quickly, almost without sample preparation steps. Most of real-time approaches are based on direct mass spectrometry (DMS), a method of analyzing samples without the need for separation or pre-treatment steps. Instead, the sample is directly introduced into the mass spectrometer for analysis. In this context, ambient ionization mass spectrometry (AIMS) techniques are widely represented and successfully used. Extractive-liquid sampling electron ionization-mass spectrometry (E-LEI-MS) represents a different analytical strategy that allows coupling ambient sampling with electron ionization (EI), avoiding any sample preparation step and providing identification based on the comparison with the National Institute of Standards and Technology (NIST) library spectra. E-LEI-MS consists of a dispositive for solvent release and sampling at ambient conditions coupled with an EI source of a single quadrupole mass spectrometer. A micromanipulator allows fine (x,y,z) positioning of a sampling tip. MS can operate in scan or SIM modes depending on the application goals and requirements. Several preliminary successful results were already obtained due to the highly informative EI mass spectra generation. The system was applied to the analysis of active ingredients in pharmaceutical tablets, pesticides on fruit peel, a drug of abuse (cocaine) determination in banknotes, and analysis of unknown components on painting surfaces. Both forensic and artwork applications allowed determining the spatial distribution of the analytes. Here we present a proof-of-concept of E-LEI-MS for targeted/non-targeted analysis and semi-quantitative detection.

## Introduction

Extractive-liquid sampling electron ionization-mass spectrometry (E-LEI-MS) is a prototype developed to combine the advantage of ambient sampling with the high identification power provided by electron ionization (EI). E-LEI-MS technology is strictly related to the direct electron ionization (DEI) LC–EI–MS interface presented by Cappiello et al., which allows the conversion of a liquid nanoflow rate to gas phase directly inside the ion source^1^. DEI was successfully employed in many applications^2^; therefore, the same setup was exploited to develop E-LEI-MS, inspired also by the liquid micro-junction surface sampling probes (CF-LMJ-SSPs) to directly sample soluble materials from surfaces for subsequent atmospheric pressure ionization sources^3,4^.

Despite E-LEI-MS being an innovative analytical approach, it refers somehow to ambient mass spectrometry, at least from the sampling point of view. Ambient MS is nowadays a fast-growing approach for real-time sampling and analysis in the native environment of various matrices without either sample preparation or chromatographic separation. With ambient MS, sample manipulation is needless or reduced to a minimum, samples can be handled in the open air and directly analyzed, thus preventing possible alterations or contaminations. Ambient MS was initiated with the development of desorption electrospray ionization (DESI)^5,6^ and direct analysis in real-time (DART)^7^. Since their first introduction, DESI and DART have been continuously improved to enhance performance and suitability in many application fields, spanning from bioanalytical to environmental, forensic, reaction monitoring, and others^8^. Atmospheric pressure ionization techniques (API), such as ESI, APCI, and APPI, are typically used in ambient MS, and jointly, various techniques based on different physical-chemical mechanisms, were developed for the direct ionization of the analytes in their original environment^9,10^. API techniques mainly produce protonated or deprotonated molecules, with or without adduct ions, thus requiring MS-MS or high-resolution MS for analyte identification and characterization. These ionization techniques, in particular ESI, are characterized by high sensitivity and robustness, however, their response is often compound-specific and can be affected by ion suppression or enhancement^6^. Unlike the above-mentioned techniques, this paper proposes a new real-time approach for analytes introduction into a standard single quadrupole mass spectrometer equipped with an electron ionization (EI) source, to increase the identification power in direct analysis. To the best of our knowledge, this is the first real-time MS technique using EI for compound ionization. Sampling occurs in ambient conditions and neither sample preparation nor manipulation is required, contrary to what is necessary for separation techniques. A suitable solvent is deposited onto the sample surface where analytes are dissolved and transferred into the EI ion source of a single quadrupole mass spectrometer by the effect of the high vacuum using a sampling tip. Right after, the MS data acquisition begins. E-LEI-MS operates at atmospheric pressure and ground potential, analytes entering the ion source in a liquid phase, are sprayed into the ion source where high-temperature and high-vacuum conditions promote their gas-phase conversion. A 70-eV electron beam effects the typical EI ionization, providing highly informative and National Institute of Standards and Technology (NIST) searchable EI mass spectra for identifying unknown compounds or detecting targeted molecules in SIM acquisition mode. Lack of chromatographic separation may lead to overlapping ion formation, but, in most cases, background ions interfere to a limited extent without preventing the identification of targeted or untargeted compounds. Gas-phase ionization provides limitless small molecule applications scarcely influenced by the matrix composition or polarity of the compounds or the extraction solvent. Being a proof of concept, preliminary data are very encouraging and demonstrate that E-LEI-MS provides robust real-time data, an essential requirement for fast screening and high throughput.

## Results and discussion

Several prototype adjustments were tried before achieving the final configuration. Several operating parameters were considered, such as capillary dimensions, aspiration rate, solvent delivery mode, type of valve used as an on/off device, and sampling mode. The optimized system configuration, described in the following section, was tested with different matrices to demonstrate its performance and versatility. Working parameters were set depending on the sample nature and application field. The application workflow was designed to demonstrate the system's capability to operate in several conditions and modes: targeted and untargeted screening, two dimensions (2D) and three dimensions (3D) analyses.

### Configuration of the E-LEI-MS system

**Figure 1:**
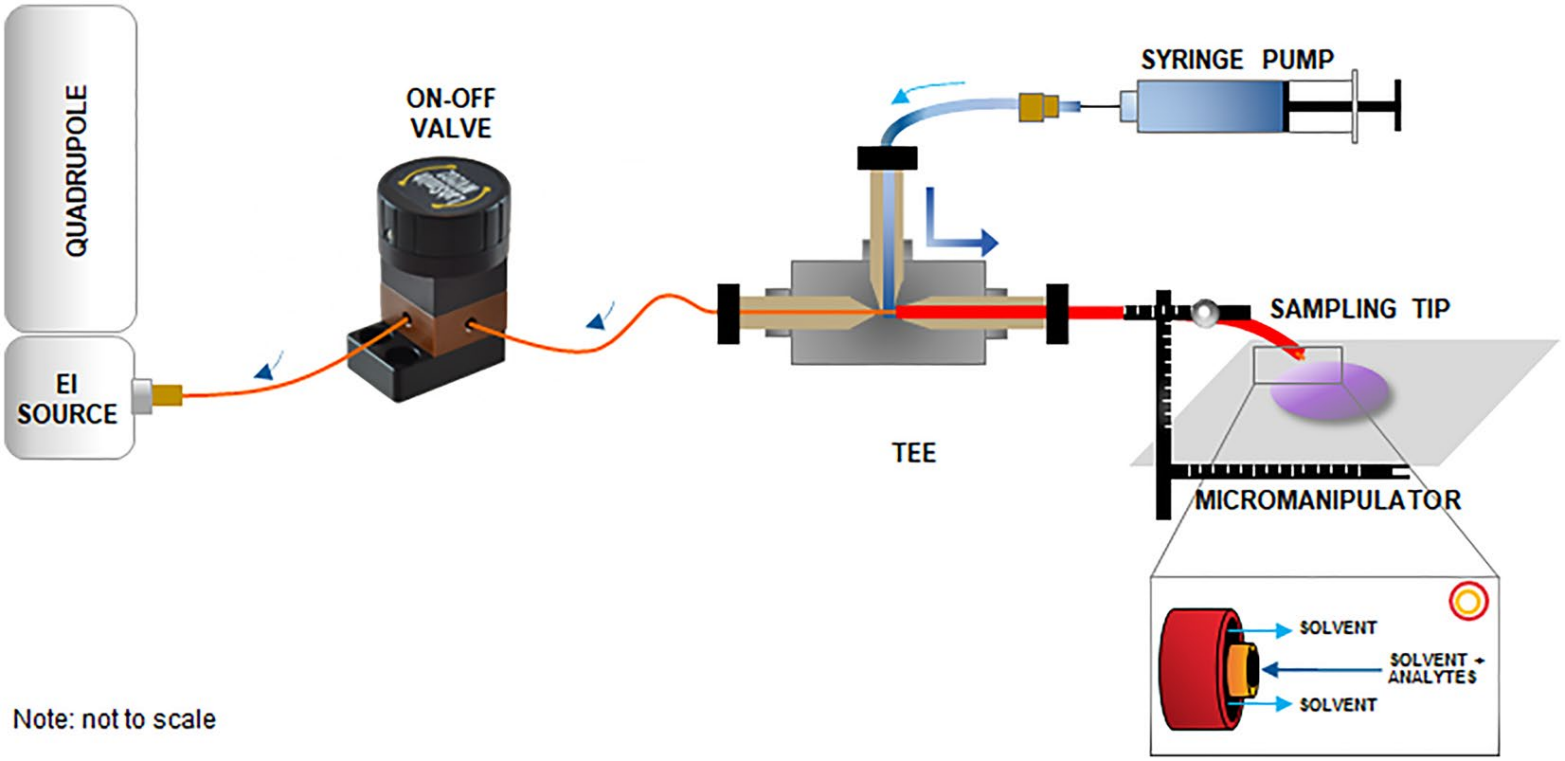
Configuration of the E-LEI-MS system.

The entire E-LEI-MS apparatus consists of a single quadrupole mass spectrometer Agilent Technologies 5975 inert Mass Selective Detector (Agilent Technologies, Santa Clara, CA, USA) equipped with an EI source, a sampling tip (fused silica capillary; 30 μm I.D; 375 μm O.D.; Polymicro Technologies (Phoenix, USA), an MV201 manual microfluidic 3-port valve, 2 positions (+ closed), “L” pattern flow, valve volume 170 nL (LabSmith, Livermore, CA, USA), a tee connector, a capillary for solvent delivery (peek tube; 450 μm I.D.; O.D. 660 μm; 10 cm length), and a micromanipulator (Standa, Vilnius, Lithuania). The solvent is delivered by a KD Scientific syringe pump (KD Scientific Inc., Holliston, MA, USA) equipped with a 1-mL syringe (Hamilton, Bonaduz, Switzerland), directly connected to the tee through a Teflon tubing. Figure 1 and Fig. S1 show the system configuration. The sampling tip is the E-LEI-MS core and consists of two coaxial tubings. The inner tubing (orange) is connected to the EI source passing through an on–off valve and crossing a tee up to the sampling spot. The outer tubing (red) delivers the appropriate solvent surrounding the inner tubing from the tee to the sampling spot. A syringe pump filled with the solvent is connected to the tee. When the syringe pump is activated, the solvent flows between the two tubings up to the sampling spot, where it precisely mixes with the analytes. The system vacuum effect immediately delivers the solution to the ion source through the inner tubing. The on–off valve regulates access to the ion source. The sample signal appears after approximately 1 min after valve opening. MS acquisition is turned on before valve actuation. The micromanipulator can operate on x-y-z axes and angle degrees to finely adjust the sample and tip relative position with an accuracy of 0.1 mm. The final configuration was tested by analyzing spots of caffeine and chlorpyrifos solution to test repeatability and absence of carryover phenomena with satisfactory results (Figs. S2–S4).

### Applications

#### Pharmaceuticals

**Figure 2:**
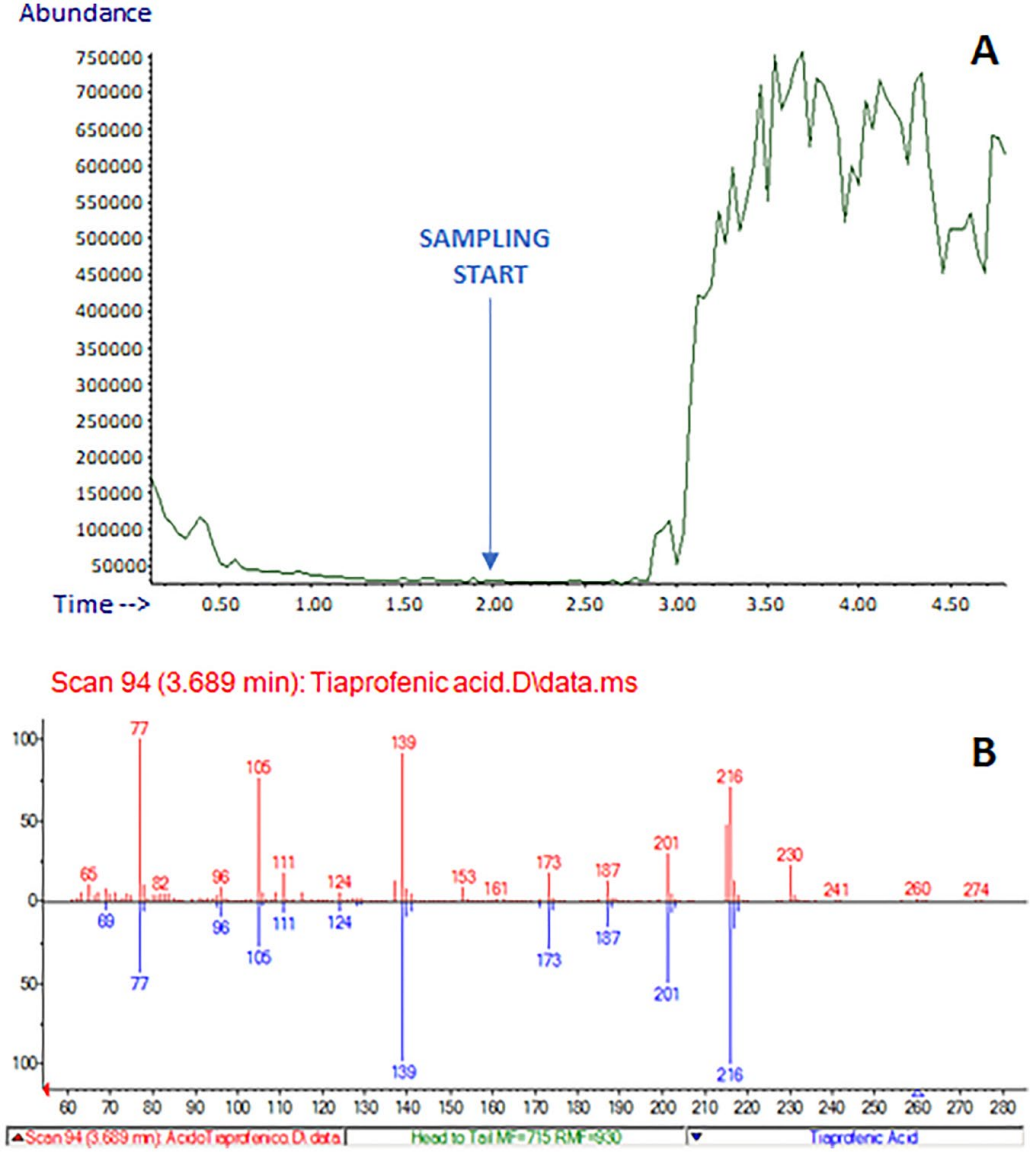
(**A**) Analysis of a Surgamyl tablet: TIC; (**B**) NIST library spectrum matching: red, experimental spectrum; blue, library spectrum. The arrow indicates the sampling start.

This application was focused on the untargeted identification of the main active ingredients in selected medicinal tablets. Tiaprofenic acid contained in Surgamyl tablets was detected and correctly identified by the NIST library in scan acquisition mode, despite the simultaneous presence of several excipients in the pharmaceutical formulation, which could adversely affect spectral similarity, therefore, compound identification. Figure 2a shows the total ion current (TIC) signal acquired during the E-LEI-MS analysis of Surgamyl, showing a signal increase at 3.00 min when the aspirated solution in acetonitrile (ACN) reached the EI source; Fig. 2b shows the tiaprofenic acid identification by the NIST library search, with a spectral match of 93.6%. The experimental spectrum was recorded at 3.7 min. A few background ions (*m/z* 153 and 230) coming from the matrix do not interfere with the compound identification.

**Figure 3:**
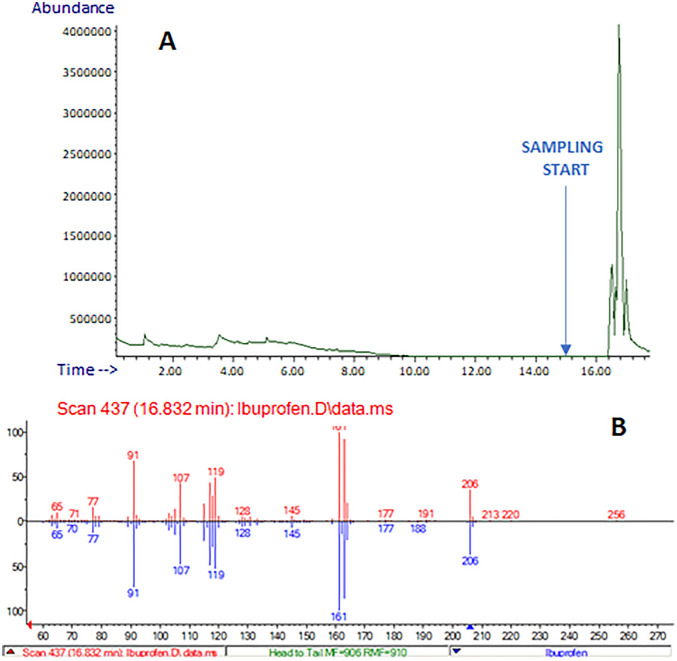
(**A**) Analysis of a Brufen tablet: TIC; (**B**) NIST library spectrum matching: red, experimental spectrum; blue, library spectrum. The arrow indicates the sampling start.

A similar experiment was carried out using Brufen tablets (Fig. 3): the TIC signal increased significantly after the sampling, and the spectral match with the NIST library resulted in the undoubted identification of the active ingredient ibuprofen dissolved in ACN. E-LEI-MS capability to identify active ingredients in commercial drugs without sample preparation was demonstrated for both types of tablets. For convenience, the MS acquisition starts before the actual sample analysis. The arrows in the figures indicate the moment the tip is positioned on the sample surface.

**Figure 4:**
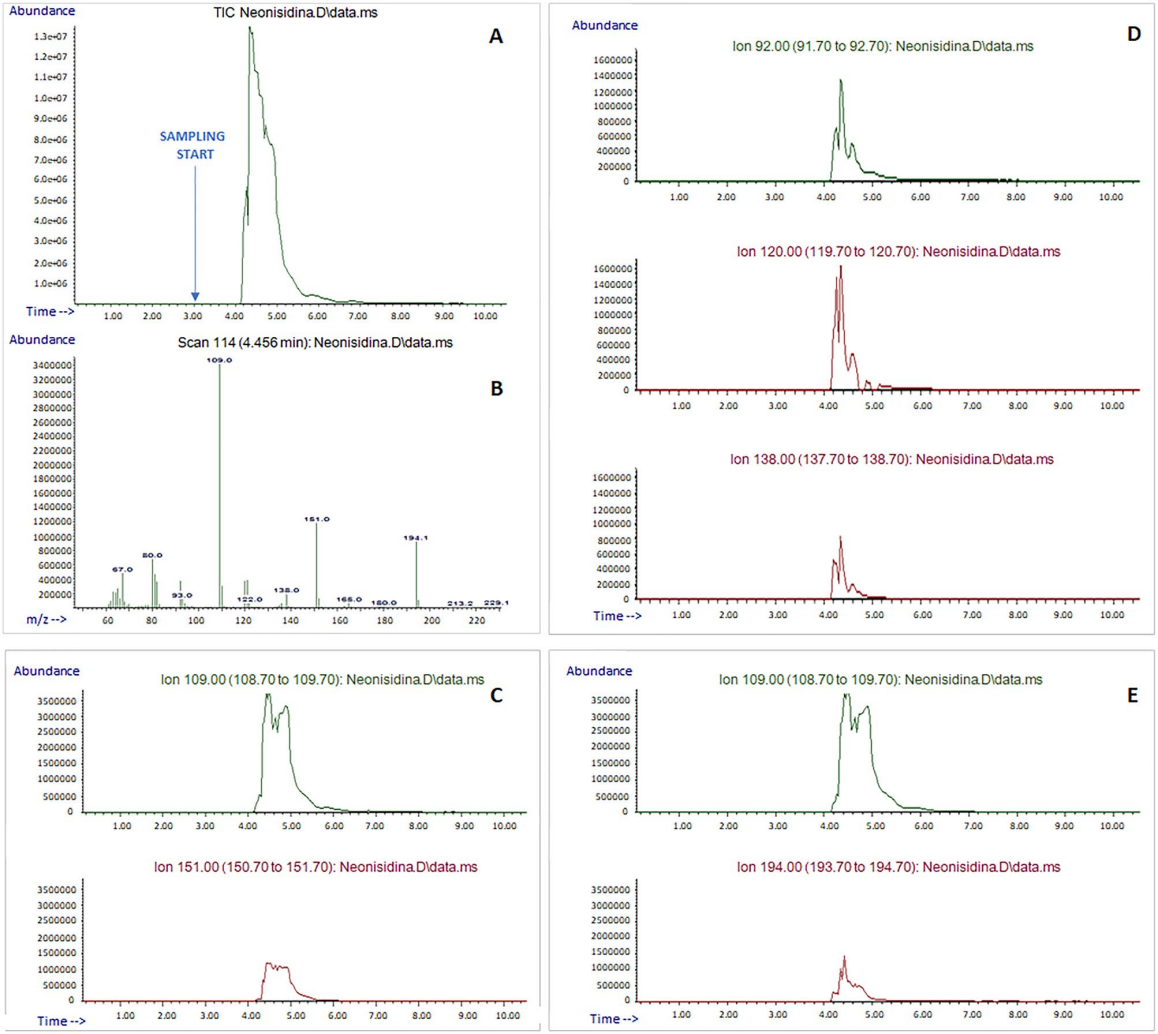
(**A**) Analysis of a NeoNisidina tablet: TIC; (**B**) EI experimental full spectrum; (**C**) acetaminophen selected ions: SIM; (**D**) acetylsalicylic acid selected ions: SIM; (**E**) caffeine selected ions: SIM. The arrow indicates the sampling start.

The analysis of NeoNisidina tablets (Fig. 4), containing acetylsalicylic acid (250 mg), acetaminophen (200 mg), and caffeine (25 mg) was carried out in scan acquisition mode, using ACN as solvent. Due to the simultaneous presence of three different targeted compounds in the same formulation, selected ions were monitored to visualize each specific signal. The selected ions were *m/z* 92, 120, and 138 for acetylsalicylic acid; *m/z* 109 and 151 for acetaminophen; *m/z* 109 and 194 for caffeine. All fragments were properly detected in SIM, demonstrating E-LEI-MS capability of identifying multiple target compounds in the same untreated matrix. These applications demonstrate E-LEI-MS potential to identify different types of pharmaceuticals, underlining its utility in quality control and forensic applications when a rapid identification of substances is needed. Compared to other efficient real-time MS technologies^7,11,12^, E-LEI-MS offers a higher identification capability because of the reproducible fragmentation pattern. This application suggests also that the future perspective of coupling E-LEI-MS to high-resolution mass spectrometry (HRMS) could be resolutive for identifying targets with the same fragments, such as *m/z* 109 for acetaminophen and caffeine, which currently limits the specific attribution of fragments. E-LEI-MS can be considered somehow a selective approach considering that salts, heavy or non-volatile molecules are not ionized in the EI source and then detected. Adducts are not formed compared with API sources, and the chosen solvent can dissolve selectively target analytes. Despite the complexity of the matrix, these experiments show clearly that EI ionizes the main component of the pharmaceutical formulations, generating spectra not affected by excipients and other interferent components. Spectra deconvolution can further increase the library match score.

### Pesticides analysis and food quality control

**Figure 5:**
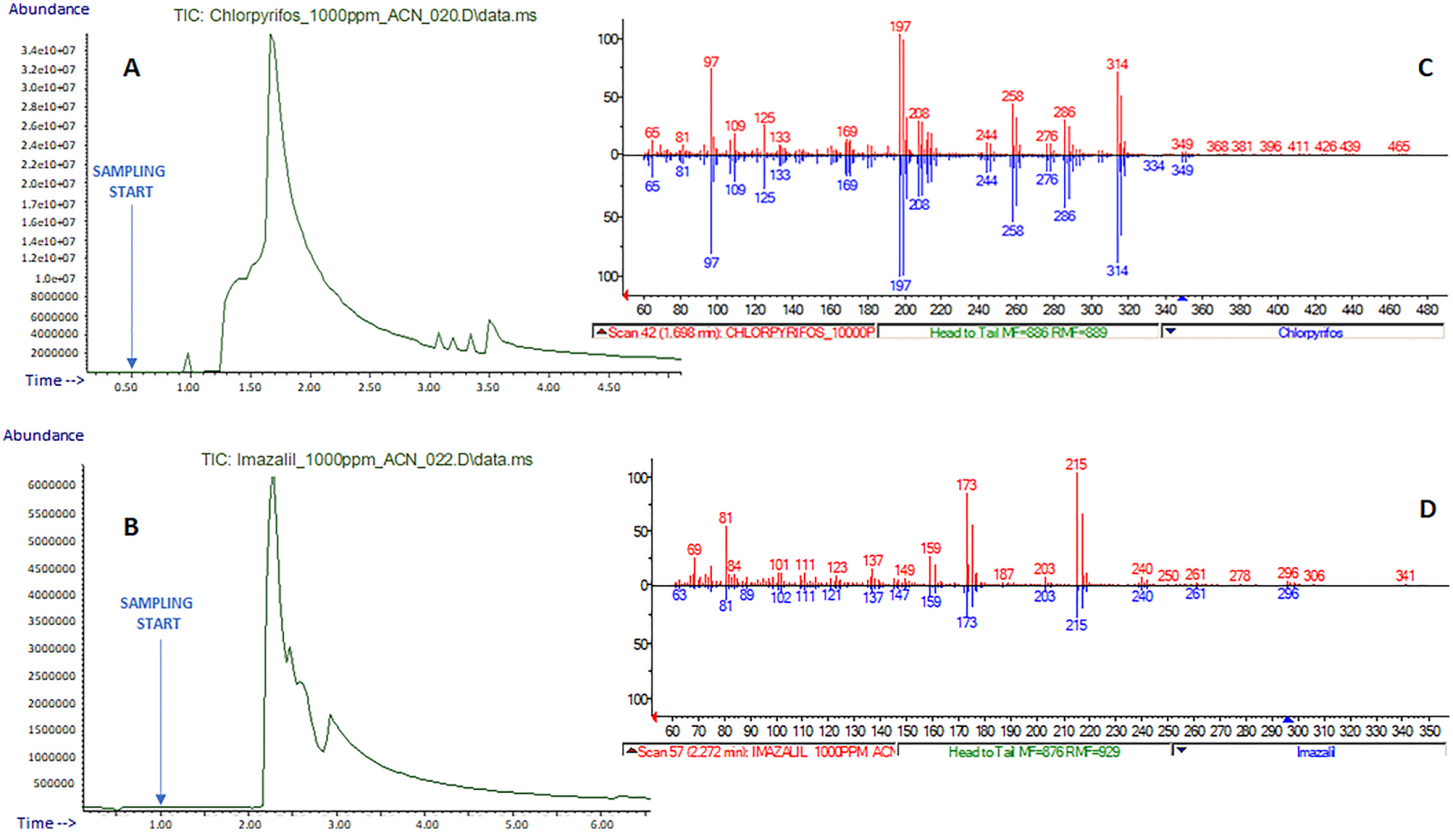
(**A**) Analysis of an orange peel fortified with chlorpyrifos: TIC; (**B**) analysis of an orange peel fortified with imazalil: TIC; NIST library spectra matching: red, experimental spectra for chlorpyrifos (**C**) and imazalil (**D**); blue, library spectra. The arrows indicate the sampling start.

A targeted determination of pesticides in fruit peels was carried out to demonstrate the applicability of E-LEI-MS in food and environmental analysis. Pesticides were selected based on their specific use on orange and banana fruits; in particular, chlorpyrifos and imazalil were used to fortify orange peels, and benomyl was used on banana peels. An orange peel specimen (3 × 3 cm, 0.5 cm thickness) was fortified with 20 μL of chlorpyrifos solution at a concentration of 1000 mg/L. The orange specimen was stored for 24 h at room temperature to allow the complete standard adsorption on the surface and solvent evaporation before analysis. The same procedure was repeated using the same volume of an imazalil solution at a concentration of 1000 mg/L. The concentrations of the two pesticide solutions were calculated to match approximately those present in common commercial formulations used in agriculture. The experimental results are shown in Figure 5. TIC signals were clearly defined, and chlorpyrifos and imazalil EI spectra were correctly identified by the NIST library with a spectral similarity of 90.3 and 97.0%, respectively, despite the presence of the matrix. A banana peel specimen (3 × 3 cm, 0.5 cm thickness) was fortified with 20 μL of a benomyl solution at a concentration of 1000 mg/L. In this case, the identification resulted in a match equal to 75.2% (Fig. S5). Following solvent evaporation, the contaminants were distributed onto an area of the peel surface of approximately one square centimeter, resulting in a pesticide distribution of approximately 20 μg/cm^2^. Moreover, the amount of standard reaching the MS is related to the sampling tip aspiration. Based on these considerations, the obtained signal and the spectral match were satisfactory.

A quantitative approach was attempted by calculating a calibration curve for chlorpyrifos. Solutions of chlorpyrifos at five concentration levels (50, 100, 500, 1000, and 2000 mg/L) were used to fortify orange peel specimens. The analyses were carried out by sampling each peel for 1 minute, and the curves were plotted based on the concentration levels (x-axis) and the recorded areas of the signals corresponding to m/z *314* ion (y-axis) (Fig. S6). The resulting equation was y = 48,302.6x − 2,371,974.6 with a coefficient of determination R^2^ of 0.9950 and completely satisfactory, considering the wide range of concentrations tested, the total absence of sample preparation, and, especially, the prototype stage of E-LEI-MS. No carryover was observed, and repeatability resulted in a coefficient of variability (CV%) within 8% for all concentration levels.

### Forensic

**Figure 6:**
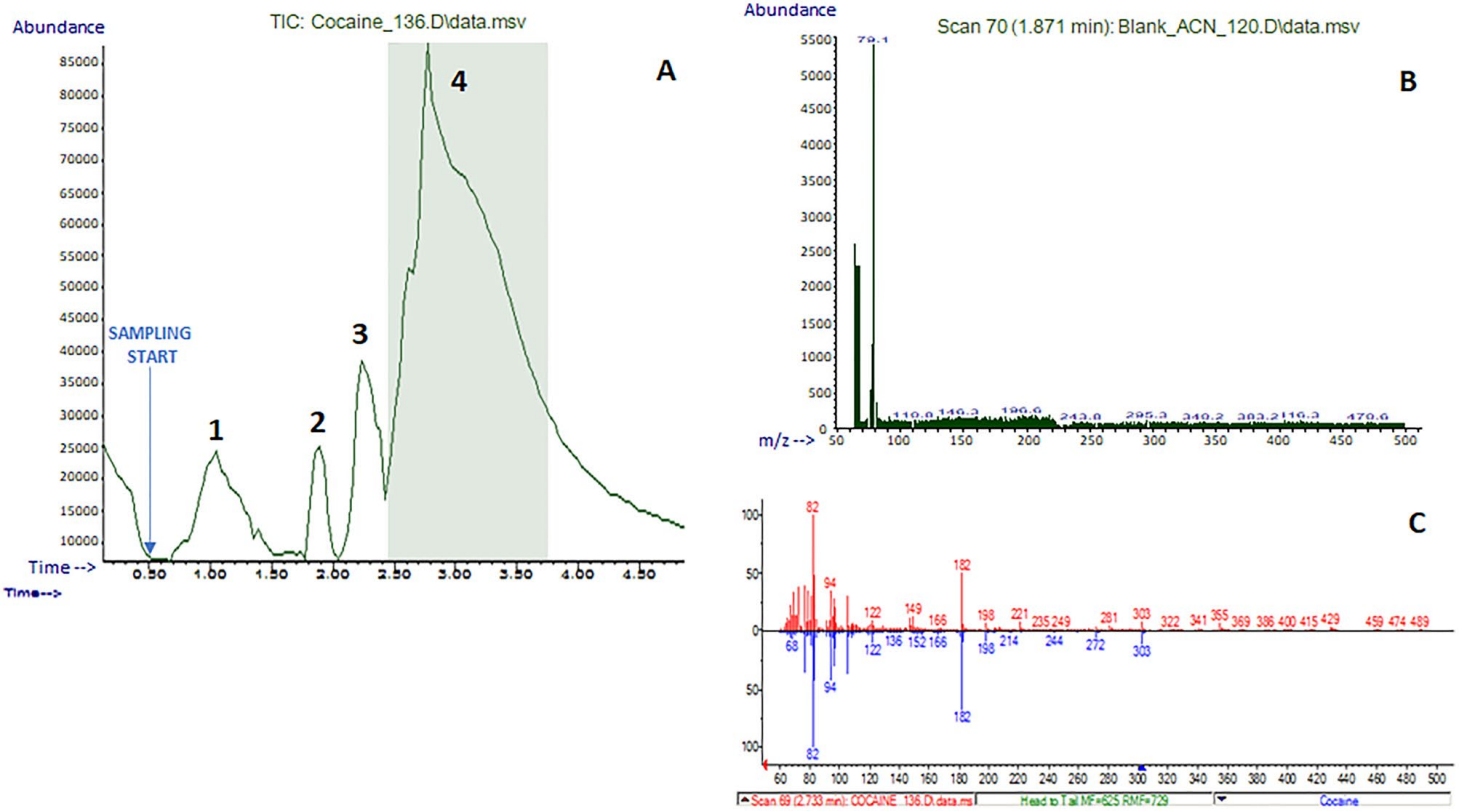
(**A**) Analysis of spots 1–3 (intact) and 4 (cocaine) on a banknote: TIC; (**B**) EI experimental full spectrum of a negative spot (matrix); (**C**) NIST library spectrum matching: red, experimental spectrum; blue, library spectrum. The arrow indicates the sampling start.

Applications based on the rapid detection of illicit drugs via direct analysis in real-time mass spectrometry are enormously increasing since the last decade^13–17^. Law enforcement needs analytical devices to enable the fast identification of drugs of abuse, due to the rapid introduction of new molecules with psychotropic effects on the illicit market that may bypass conventional control systems and protocols. Following this trend, E-LEI-MS was applied to the analysis of a cocaine-spotted banknote. These experiments aimed to provide 2-D tests to verify if the system can fast recognize specific substances on selected surface spots, compared to controls, thus rendering analytes spatial distribution. The banknote was spotted with the target solution, then dried at ambient conditions before analysis. Figure 6 shows the signals corresponding to the sampling of three negative control spots (1–3) and one (4) positive control spot corresponding to the stain of 20 μL of a cocaine solution at a concentration of 100 mg/L on a 1 cm^2^ spot.

The signal intensity corresponding to the cocaine-fortified spot (4) increased immediately after the aspiration, decreasing after 2 min, highlighting the system’s capability to provide fast results without carryover. The cocaine EI spectrum was unambiguously identified by matching the NIST library with a high spectral similarity score (> 90%). In these experiments, E-LEI-MS demonstrated an unparalleled identification power which is mandatory in forensic applications and represents the main point of strength of this approach.

### Artwork analysis—paintings

The potential of E-LEI-MS to identify completely unknown components in a complex matrix was tested in artwork analysis, particularly in detecting untargeted compounds in paintings for compounds attributable to the type of colors or fixative used, which helps select the proper materials in restoration interventions. An accurate understanding of the materials used in a particular painting is also fundamental for attributing the artwork to an artist or a historical period. Acrylic paintings on canvas were provided by the “Scuola di Conservazione e Restauro” laboratories of the University of Urbino.

**Figure 7:**
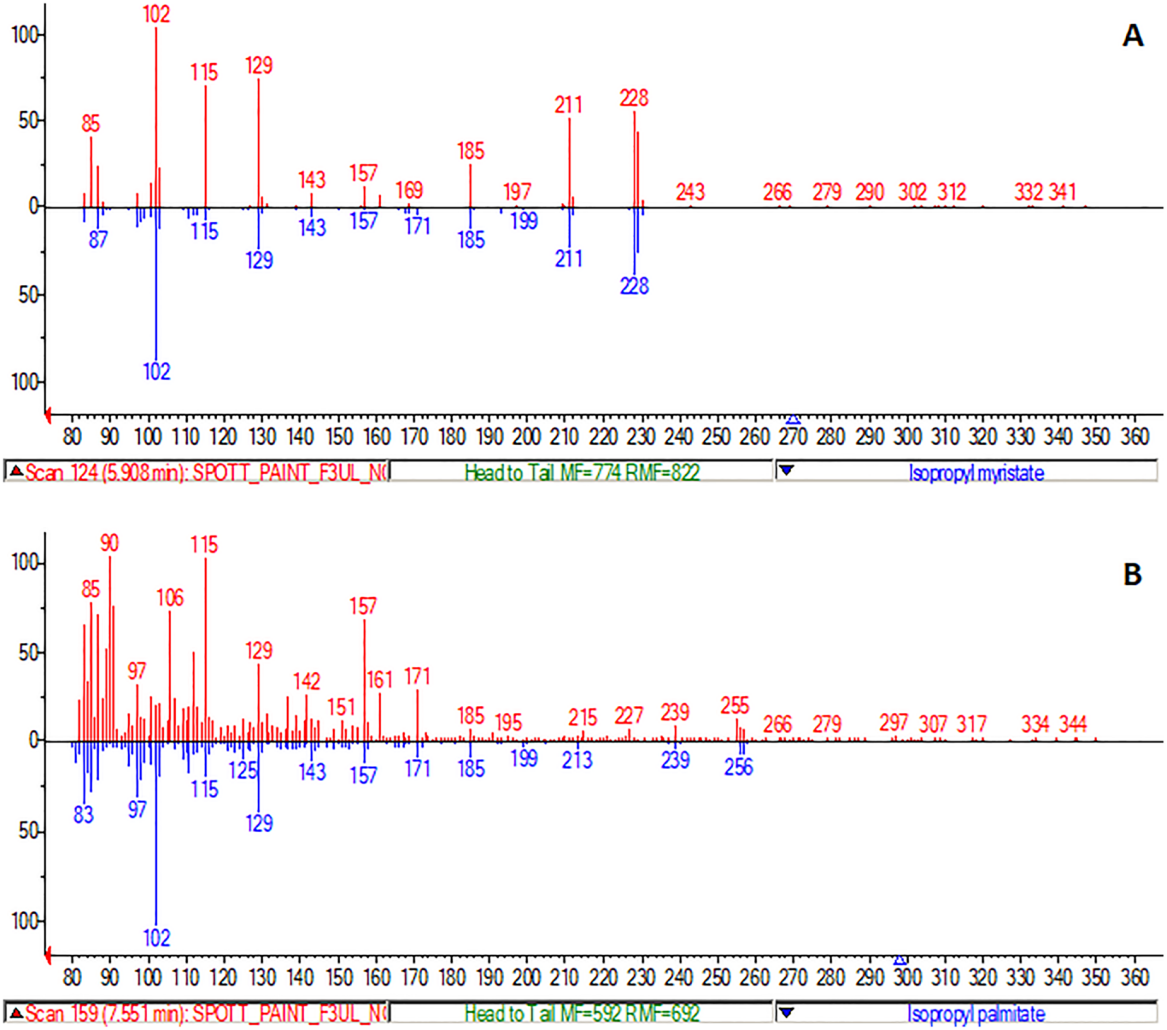
(**A**) Spectrum obtained from the analysis of the painting surface: TIC; (**B**) spectrum obtained from the analysis of the painting deeper layer: TIC. NIST library spectra matching: red, experimental spectra for isopropyl myristate (**C**) and isopropyl palmitate (**D**); blue, library spectra.

The study aimed to identify painting surface components (e.g., synthetic or natural fixatives); this information plays a fundamental role in preparing the proper restoration procedure. A system capable of providing these data by analyzing a small painting surface demonstrates great potential in this application field. For these reasons, several solvents were tested on small squares of painting samples: water, ACN, MeOH, dichloromethane (CH_2_Cl_2_), hexane, and octanol. ACN was selected for the combination of proper dissolving power and viscosity. This study aimed to investigate also E-LEI-MS's ability to perform unknown and multilayer analyses permitting the sample mapping in three dimensions, where the third dimension corresponds to the deeper layers. For this reason, the sample was exposed to ACN for a longer time allowing the in-depth extraction of the compounds. Sampling started after 2 min of solvent release from the tip. As shown in Fig. 7, the first spectrum at 5.91 min corresponded to isopropyl myristate, with a NIST spectral similarity of 93.4%. This result is satisfactory, considering the matrix complexity, which could affect the spectrum quality by limiting unknown compounds’ identification. The identified compound was concordant with the sample nature; isopropyl myristate is an esterified fatty acid, potentially a component of fixative varnish applied on paintings. After another three minutes of sampling in the same surface area, another spectrum was acquired corresponding to isopropyl palmitate (spectral similarity 43.2%), also used as a fixative component. Isopropyl palmitate was detected later than isopropyl myristate, probably because present in a deeper layer of the painting; therefore its extraction required a longer time. This application clearly shows the E-LEI-MS identification power of unknown compounds since it was conducted using a matrix whose surface components were totally unknown. As a micro-invasive technique, E-LEI-MS is particularly suitable for this kind of application when preserving prestigious matrices is mandatory.

### Greenness evaluation

The Greenness evaluation of E-LEI-MS was performed using the Analytical GREEnness Metric Approach, AGREE^18^, which was recognized as one of the most applicable to many analytical procedures^19^. AGREE is based on 12 criteria at which is attributed a default value equal to 2, which represents the impact of the single parameter on the entire procedure. The default weight value can be changed from 1 to 4, to make the evaluation tool flexible and suitable for each technology. AGREE evaluation of E-LEI-MS was based on the different experiments performed. Figure S7 shows the results obtained, maintaining the default value for all criteria. A very satisfactory score of 0.79 (out of 1) was obtained, showing the greenness of the new system. As clearly visible in Fig. S7, the lowest score, in red, was attributable to the positioning of the analytical device, which at the present, does not allow on-site analysis. In this view, because E-LEI-MS is a simple device, further developments will be aimed to make it applicable to a portable device, thus increasing enormously its ecological potential.

White analytical chemistry (WAC)^20^ is a new concept that is worth to be mentioned here. In brief, WAC is based on the idea that increasing the greenness of an analytical method or technology must not undermine its functionality. E-LEI-MS was proved to be a fast and environmentally friendly ambient MS approach, with a high identification power guaranteed by EI, compared to conventional ambient MS techniques, in the view to meeting the principle of WAC mentioned above.

## Methods

### Materials

LC grade solvents, methanol (MeOH), acetonitrile (ACN), dichloromethane (CH_2_Cl_2_), hexane, and octanol were purchased from VWR BDH Chemicals HiPerSolv Chromanorm (Milan, Italy). Milli-Q water was produced using a Millipore Direct-Q 3 UV purification system (Millipore Corp., Milan, Italy). Fused silica capillaries of various internal (I.D.) and external (O.D.) diameters were purchased from Polymicro Technologies (Phoenix, USA).

Pesticides: chlorpyriphos (CAS 2921-88-2; PESTANAL, analytical standard 99.9% purity), imazalil (CAS 35554-44-0; PESTANAL, analytical standard 99.8% purity), and benomyl (CAS 17804-35-2; PESTANAL, analytical standard, ≥ 98.0% purity) were supplied by Sigma Aldrich. Imazalil and benomyl stock solutions were prepared gravimetrically at 1000 mg/L concentration in ACN. Chlorpyrifos stock solution was prepared gravimetrically at a concentration of 1000 mg/L in ACN. Solutions of chlorpyrifos at 50, 100, and 500 mg/L were prepared by diluting the stock solution with the same solvent.

Cocaine (CAS 50-36-2; Cerilliant, Certified Reference Material) stock solution at a concentration of 1000 mg/L in ACN was provided and manipulated by the Toxicology Laboratory A.S.T. AV1, (Pesaro, Italy).

Caffeine (CAS 58-08-2; analytical standard > 99.0% purity) solution was prepared gravimetrically at 1000 mg/L concentration in ACN.

### Samples

The anti-inflammatory drugs, purchased at a local pharmacy, were the following: Surgamyl produced by Scharper S.p.a (Milano, Italy) containing tiaprofenic acid (300 mg); Brufen produced by Mylan S.p.a. (Milano, Italy) containing ibuprofen lysine salt acid (200 mg); and NeoNisidina produced by Pharmaidea S.r.l. (Travagliato, BS, Italy), containing acetylsalicylic acid (250 mg), acetaminophen (200 mg), and caffeine (25 mg). All the tablets also contained several excipients.

Oranges and bananas were bought at a local market and were fortified by adding pesticide standards on the peel at a specific concentration. The banana peel was fortified with benomyl (20 μL of a 1000 mg/L standard solution). In two different experiments, the orange peel was fortified with 20 μL of chlorpyrifos and 20 μL of imazalil, both at a concentration of 1000 mg/L. Chlorpyrifos was added to the orange peel at increasing concentrations spanning from 20 to 1000 mg/L.

A 5-euro banknote (62 × 120 mm) was signed with circles to perform 2D and 3D experiments. Some circles were wetted with ACN and represented the negative control, whereas others were spotted with 20 μL of a cocaine solution at 100 mg/L as positive controls.

Paintings were kindly provided by the “Scuola di Conservazione e Restauro” laboratories, of the University of Urbino. Water, ACN, MeOH, CH_2_Cl_2_, hexane, and octanol were tested to select the most suitable solvent for dissolving the painting sample surface.

The flow of solvent (ACN) delivered by the syringe pump on the sample surfaces was set at 3 μL/min for all the experiments.

### Analytical conditions

ACN was used in most applications to dissolve the analytes on the sample surface and was delivered by the syringe pump at a flow rate of 3 μL/min. The timing of sampling was 1 min for all experiments and the time between two consecutive analyses or analyses of two consecutive spots was set based on the experimental time needed for reducing the signal to the baseline.

MS tuning was performed daily at an ion source temperature of 280 °C, using perfluorotributylamine (PFTB) as a reference compound, monitoring its characteristic ions. No mobile phase was admitted into the source during this procedure. The ion source can operate up to 350 °C depending on the nature, molecular weight, and boiling point of the selected compounds. The single quadrupole mass spectrometer was operated at 150 °C. The solvent and analytes aspirations were ensured by the instrument’s high vacuum pumps.

MS data acquisition was carried out in scan and SIM modes. Scan analyses were conducted in an *m/z* range of 60–500, depending on the solvent used, with a sampling rate of 0.43 scan/s, and a threshold of 10. The low sampling rate is needed for obtaining a continuous signal that does not impose a fast scan speed, as it is required in the case of chromatographic peak acquisition. Depending on the targeted molecules, SIM analyses were conducted by selecting two or three specific ions. Typical signal stabilization after sampling lasted less than a minute and did not require fast scanning rates.

The background was subtracted from all spectra before the search in the NIST library.

Blank analyses were performed after each acquisition.

Enhanced ChemStation MSD E.02.00.493 (Agilent Technologies) software was used for data acquisition and processing.

### Greenness evaluation

AGREE green assessment tool^21^ was used to evaluate the environmentally friendly character of E-LEI-MS.

### Plant sample statement

The authors declare that the collection of plant material complies with relevant institutional, national, and international guidelines and legislation. No plants and seeds were collected and used in this study. No wild species and species at risk of extinction were used in this study. Bananas and oranges were bought in a local market and only their peels were used for the experiments.

## Conclusions

E-LEI-MS, a new system for direct analysis in EI mode, is presented herein for the first time.

E-LEI-MS is an innovative and competitive real-time technique based on ambient sampling and EI-MS, which ensures a fast and reliable analytes identification.

The system allows the rapid analysis of targeted and unknown compounds without sample manipulation or preparation, as required for separation techniques, demonstrating great potential and versatility in several application fields. Satisfactory results were obtained with different matrices and samples, in 2D and multilayer analyses, highlighting the possibility of using E-LEI-MS in surface/spatial analysis. E-LEI-MS is also an environmentally friendly approach, as demonstrated by AGREE evaluation and further studies will be aimed at increasing E-LEI-MS performance and making it a portable device, with consequent improvements in the system greenness and applicability.

## Supplementary Information

Supplementary Figures.

## Data Availability

Raw data used in the generation of Figs. 2–7, S2–S4 were submitted as separate files but are not publicly available due to the prototypal nature of the system herein presented and the specificity of software needed for data processing (Enhanced ChemStation, MSD ChemStation E.02.00.493 Copyright 1989-2008 Agilent Technologies, Inc.) but are available from the corresponding author upon reasonable request.
