# Supplementary material for: Extractive-liquid sampling electron ionization-mass spectrometry (E-LEI-MS): a new powerful combination for direct analysis
Source: Sci Rep. 2023 Apr 20;13:6429. doi: 10.1038/s41598-023-33647-5 (PMC10119378; doi:10.1038/s41598-023-33647-5)
Supplement: Supplementary file 1 — Supplementary Figures. [file 41598_2023_33647_MOESM1_ESM.pdf]

Extractive-Liquid Sampling Electron Ionization-Mass Spectrometry (E-LEI-MS): a New Powerful Combination for Direct Analysis

Adriana Arigò[1,*], Giorgio Famiglini[1], Nicole Marittimo[1], Marco Agostini[2+], Caterina Renzoni[2+], Pierangela Palma[1,3], and Achille Cappiello[1,3]

[1]University of Urbino Carlo Bo, Department of Pure and Applied Sciences, LC-MS Lab, Piazza Rinascimento 6, Urbino, 61029, Italy
[2]Laboratorio di Tossicologia A.S.T. AV1, Via Lombroso 15, Pesaro, 61122, Italy
[3] Department of Chemistry, Vancouver Island University, B360-R306 – 900 Fifth St., Nanaimo, BC, Canada
[+]these authors contributed equally to this work
adriana.arigo@uniurb.it

Supplementary information

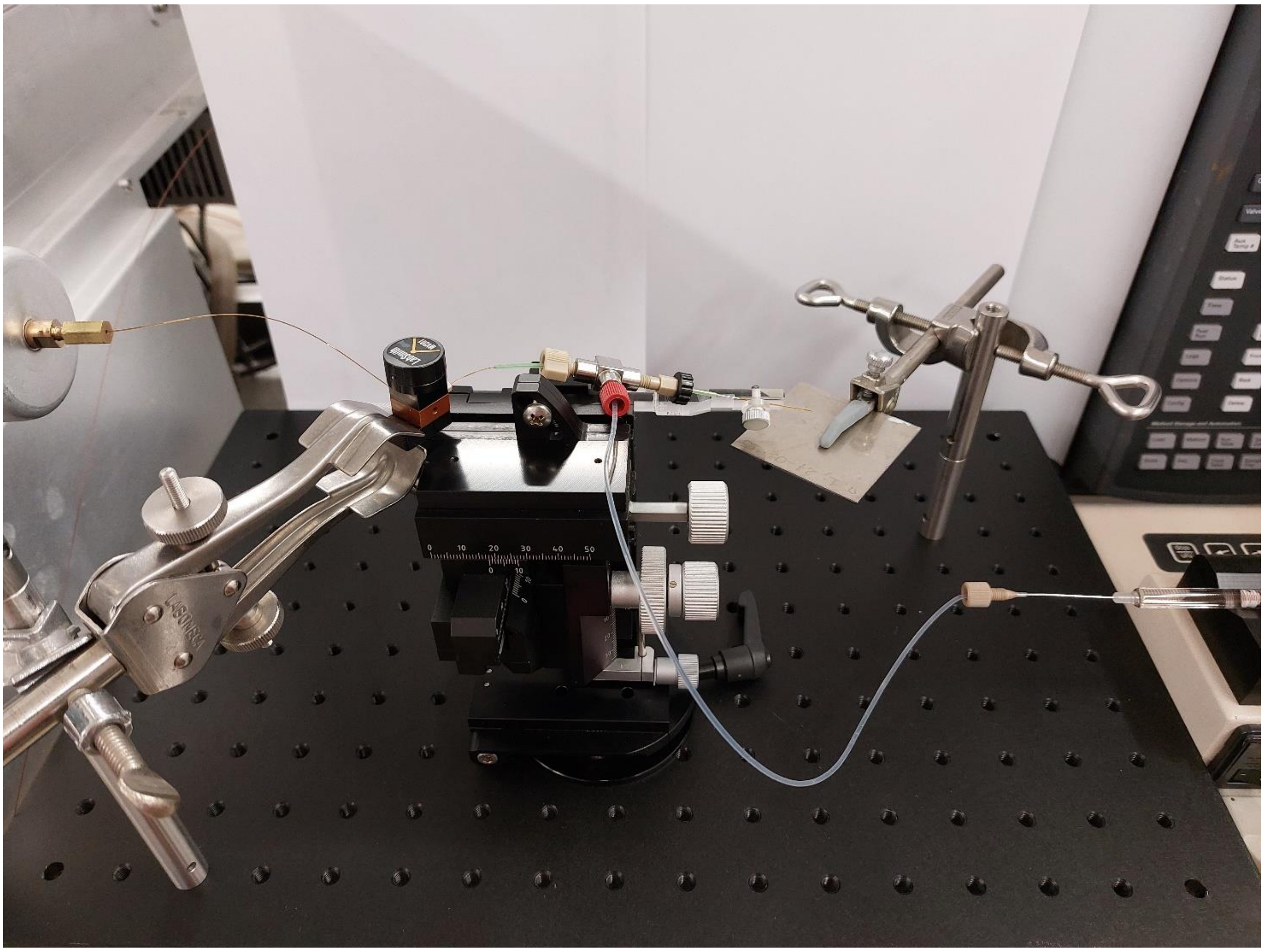

Figure S1. Picture of E-LEI-MS system.

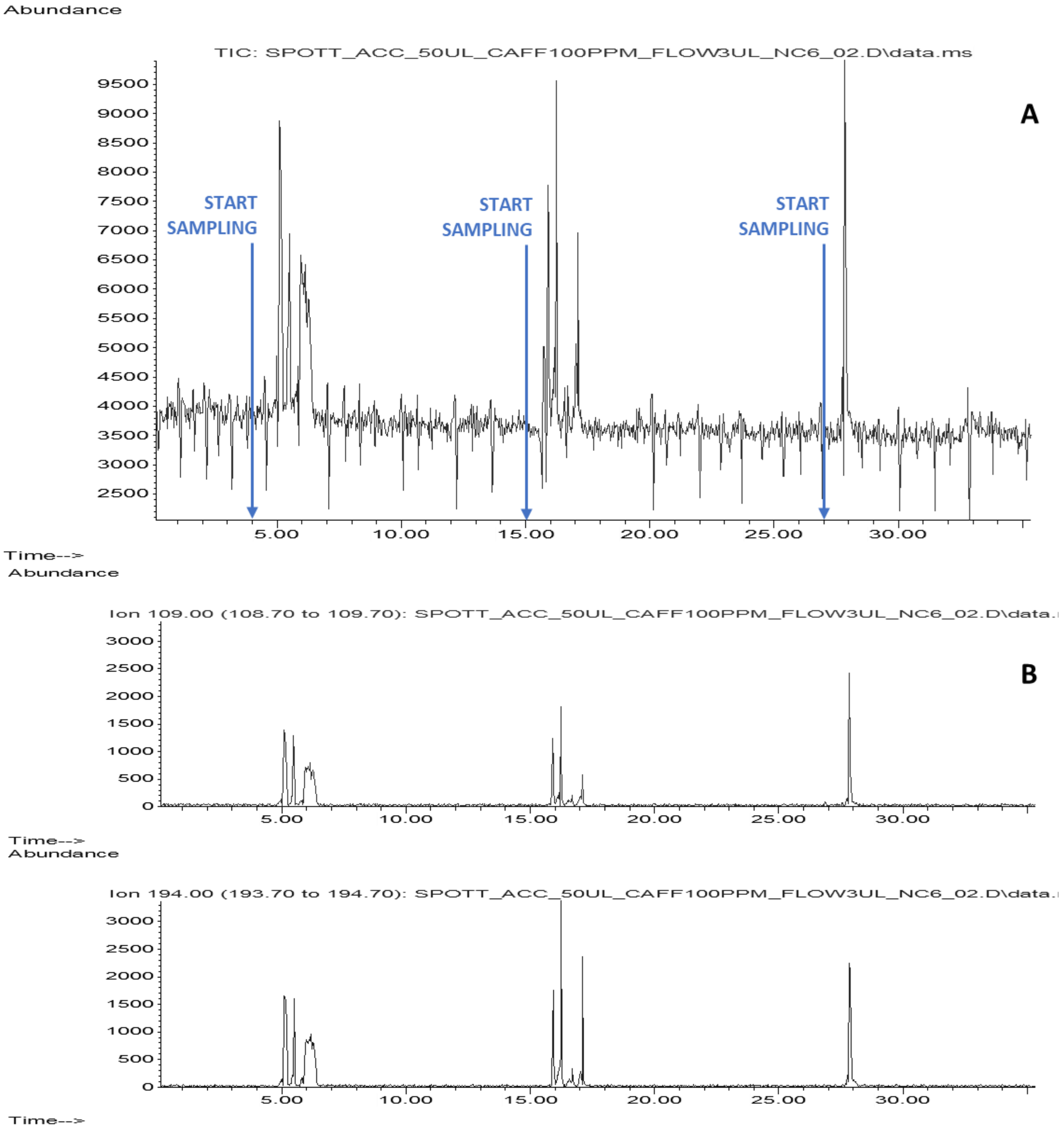

Figure S2. Analysis of caffeine spots to repeatability and of carryover tests. (A) Analysis of three consecutive spots of caffeine (50 µL of a caffeine solution 100 mg/L; sampling 30’’): TIC; (B) extracted ions m/z 109 and m/z 194. The signal decreased to the baseline within 2 min and no carryover was observed between two consecutive spots. The arrows indicate the sampling start.

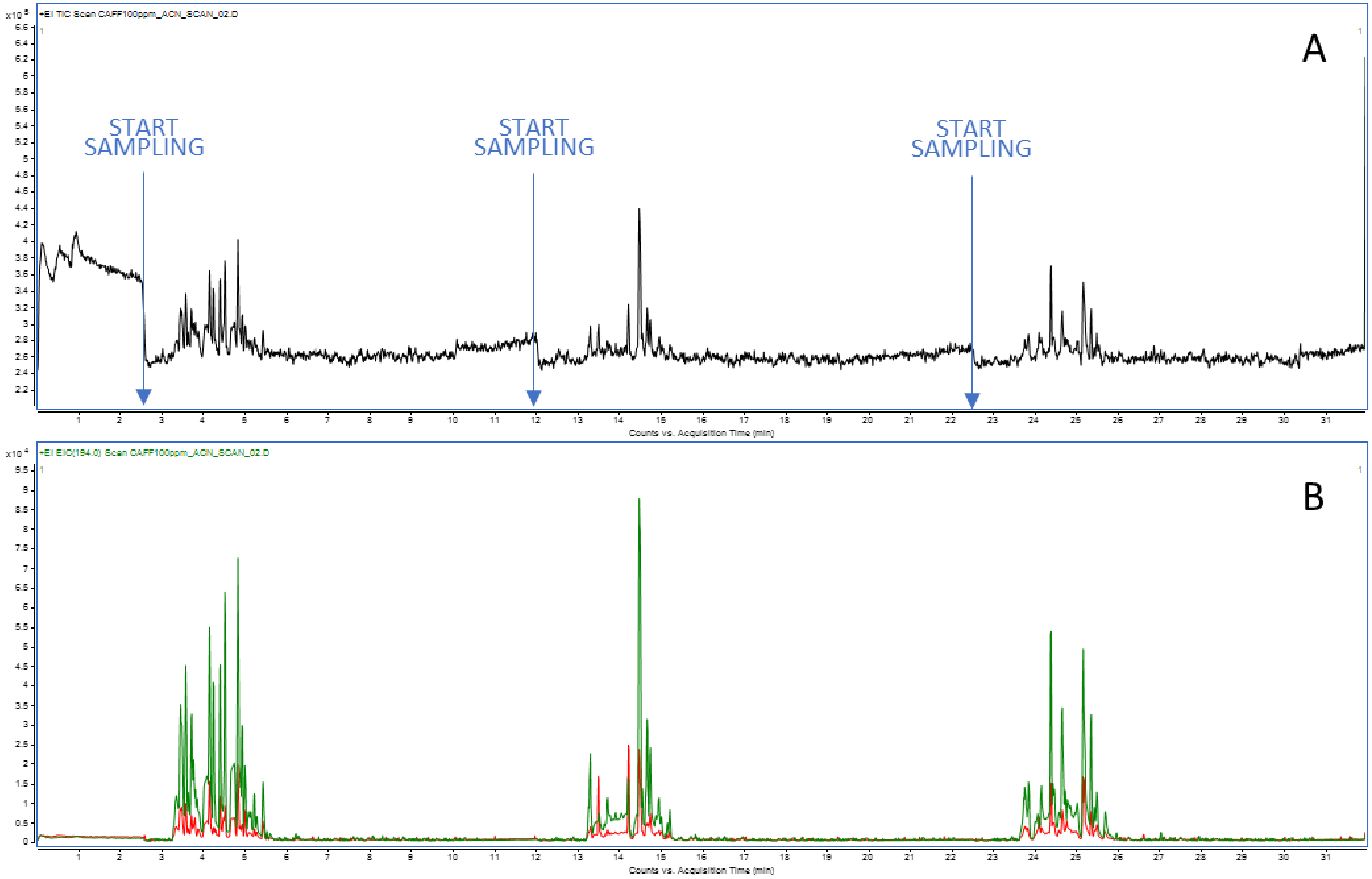

Figure S3. Analysis of caffeine spots to repeatability and of carryover tests. (A) Analysis of three consecutive spots of caffeine (50 µL of a caffeine solution 100 mg/L; sampling 90’’): TIC; (B) extracted ions m/z 109 and m/z 194. The signal decreased to the baseline within 2,5 min and no carryover was observed between two consecutive spots. The arrows indicate the sampling start.

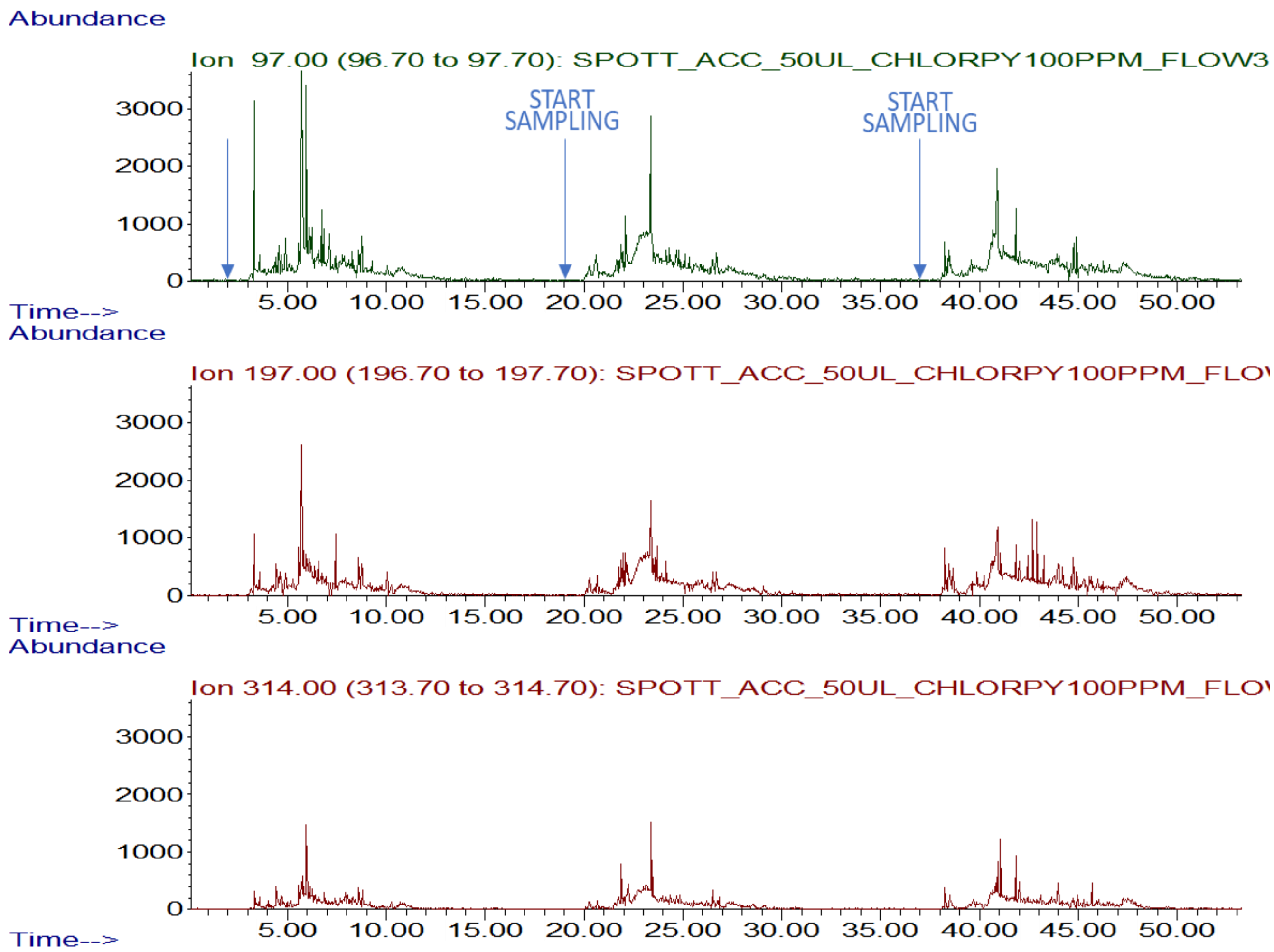

Figure S4. Analysis of chlorpyrifos spots to repeatability and of carryover tests. (A) Analysis of three consecutive spots of chlorpyrifos (50 µL of a chlorpyrifos solution 100 mg/L; sampling 30''): TIC; (B) extracted ions m/z 197 and m/z 314. The signal decreased to the baseline within 2 min and no carryover was observed between two consecutive spots. The arrows indicate the sampling start.

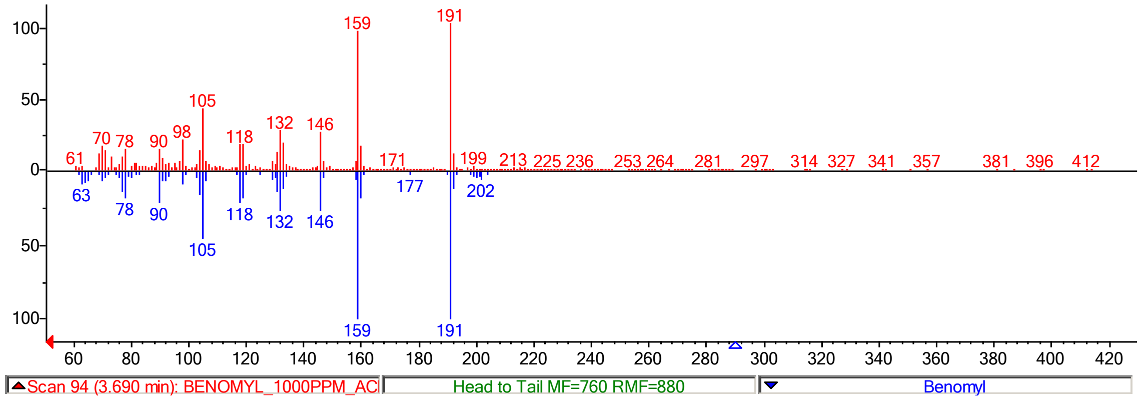

Figure S5. NIST library spectra matching: red, experimental spectrum for benomyl; blue, library spectrum.

Table S1. Chlorpyrifos data for the calibration curve. In the x-axis: the solution concentration (mg/L); in the y-axis: the signal area.

x	y
20	324333
50	906887
100	1314594
500	17518101
1000	49385264
2000	93589464

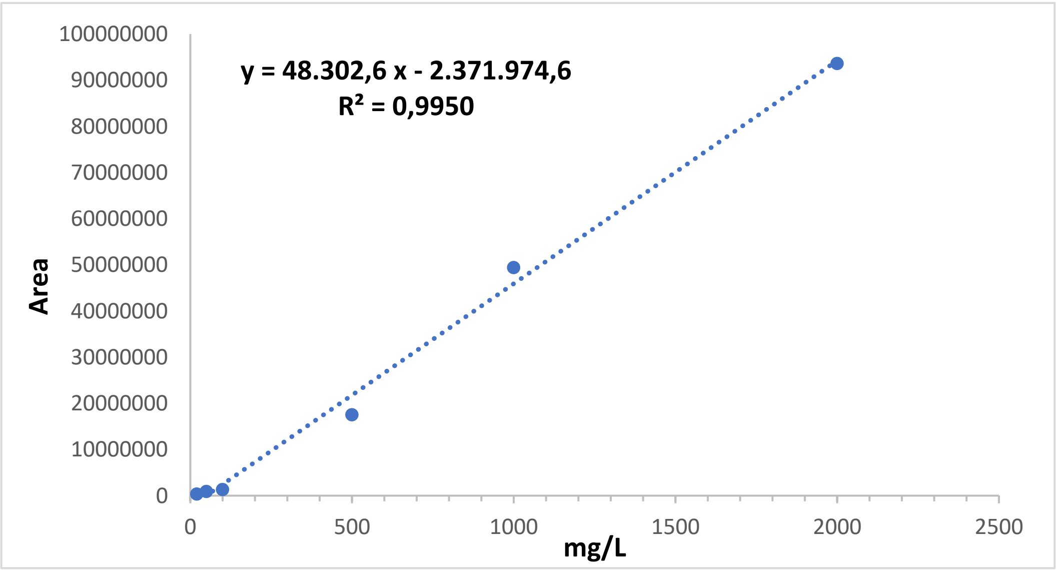

Figure S6. Chlorpyrifos calibration curve.

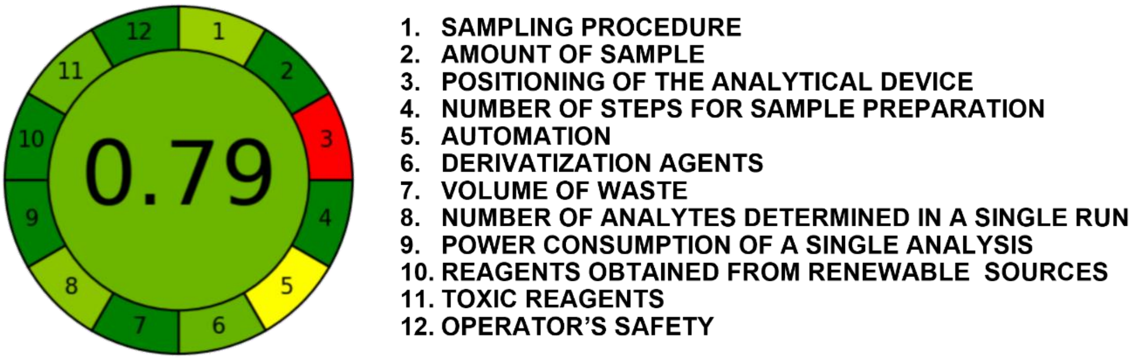

Figure S7. AGREE pictogram of the E-LEI-MS score. On the right side of the figure, the 12 criteria of AGREE are reported.
